# Diagnostic accuracy of 4D-MRA for the detection and localization of spinal dural arteriovenous fistulas: A retrospective 10-year cohort study

**DOI:** 10.1016/j.bas.2024.104176

**Published:** 2024-12-31

**Authors:** Frederick J.A. Meijer, Vincent Raymaekers, Sjoert A.H. Pegge, Jeroen HD. Boogaarts

**Affiliations:** a Department of Medical Imaging, Radboud University Medical Center, Nijmegen, the Netherlands; b Department of Neurosurgery, Antwerp University Hospital, Edegem, Belgium; c Department of Neurosurgery, Radboud University Medical Center, Nijmegen, the Netherlands

**Keywords:** Spine, Neurovascular, Magnetic resonance imaging, Angiography

## Abstract

**Research question:**

The goal of this study was to assess the diagnostic accuracy of spinal time-resolved contrast-enhanced MR angiography (4D-MRA) for the detection and localization of spinal dural arteriovenous fistulas (SDAVF) in our institution.

**Material and methods:**

Single center retrospective cohort study of patients with the clinical suspicion of a SDAVF. Patients were included who had undergone spinal 4D-MRA in the period January 2010–February 2021. A subgroup was identified, who had subsequent digital subtraction angiography (DSA), or clinical/radiological follow-up. Diagnostic performance measures of 4D-MRA were calculated (sensitivity, positive predictive value, specificity, and negative predictive value) with DSA serving as the reference standard.

**Results:**

Overall, 120 patients with the clinical suspicion of having a SDAVF and who underwent spinal 4D-MRA were identified. A subgroup of 30 patients had both 4D-MRA and DSA, or follow-up. In this group, the sensitivity of 4D-MRA for the detection of a SDAVF was 100% and specificity was 91% (positive predictive value of 95% and a negative predictive value of 100%). Sidedness was correctly identified on 4D-MRA in 74% of the cases and the level of the SDAVF in 68%.

**Discussion and conclusion:**

The results indicate that 4D-MRA has a high sensitivity and specificity for the detection and localization of a SDAVF, which is in line with previous studies published in literature. Furthermore, 4D-MRA can serve to guide DSA and shorten the procedural time, which reduces the risk of angiography related complications, and decreases patient discomfort.

## Highlights

• Spinal 4D-MRA has a high accuracy for the detection and localization of a SDAVF.
• Angiography should still be considered in case of a positive, or inconclusive 4D-MRA.
• Spinal 4D-MRA can serve to guide angiography and to shorten the procedural time.

## 1 Introduction

A spinal dural arteriovenous fistula (SDAVF) is a vascular anomaly where an abnormal connection forms between a radicular artery and a radicular vein, typically located at the dura of a nerve root sleeve (Koch, 2006). This condition causes arterial blood, under high pressure, to flow directly into the veins, leading to venous expansion and congestion. This results in spinal cord edema, which can cause neurological dysfunction and present clinically as a spinal cord lesion (Kataoka et al., 2001). Early diagnosis is crucial for timely surgical, or endovascular treatment.

Digital subtraction angiography (DSA) is the gold standard for diagnosing and evaluating the detailed angioarchitecture of a SDAVF. While DSA provides this detailed angioarchitecture, it is an invasive and labor-intensive procedure. It requires placing the patient under (local) anesthesia and inserting an x-ray guided catheter with multiple intra-arterial injections of a contrast agent at various levels of the spinal arterial tree. This exposes the patient to radiation, anesthesia, and potential procedural complications such as dissection, thrombosis, and contrast-induced (nephro)toxicity (Kieffer et al., 2002; Forbes et al., 1988). Additionally, if the entire spinal vasculature needs to be visualized, the procedure can be lengthy. Time-resolved contrast-enhanced MR angiography (4D-MRA) can improve the diagnostic accuracy of spinal MRI for detecting and localizing SDAVFs, and can identify the arterial feeder(s) and draining vein(s) (Wójtowicz et al., 2023; Raymaekers et al., 2024). Unlike invasive angiography, MRA does not carry the risk of angiography-related complications, or ionizing radiation.

This study aims to assess the diagnostic accuracy of spinal 4D-MRA for the detection and localization of a SDAVF in our own clinical practice over a period of 10 years. In this article 4D-MRA refers to both time-resolved and contrast enhanced MRA imaging.

## 2 Materials and methods

### 2.1 Study design

A single-center retrospective study was performed in a cohort of patients suspected of a SDAVF between January 2010 and February 2021. Informed consent was waived by the medical ethical committee, due to the retrospective study design. The study was conducted in accordance with the ethical standards of the 1964 declaration of Helsinki and its later amendments.

#### 2.1.1 Study population

Patients were identified by searching the local radiology PACS system for spinal 4D-MRAs performed for the clinical suspicion of having a SDAVF, between the January 2010 and February 2021. A subgroup of patients was identified, who had either both 4D-MRA and DSA, or clinical/radiological follow-up. Radiological follow-up consisted of repeat spinal MRI, while clinical follow-up involved monitoring symptom progression and conducting repeated clinical examinations, with documentation indicating the absence of SDAVF, or the establishment of an alternative diagnosis.It was confirmed that there was no treatment, or spinal surgery between 4D-MRA and DSA, or during clinical follow-up. Demographics of this study population were collected and stored in a protected database (CastorEDC). These data included mean delay time between 4D-MRA and DSA, sidedness and level of the SDAVFs, treatment after diagnosis, and the follow-up modality. 4D-MRA and DSA images were reviewed by two senior neuro-(interventional)radiologists with more than 20 years of experience in the diagnosis and treatment of neurovascular diseases.

### 2.2 Spinal MRA

Spinal contrast enhanced 4D-MRA was performed on a 3T MRI system (Siemens Trio, Erlangen, Germany). A dose of 10 ml Gadovist was injected using an automated injector (Ulrich Medical, Ulm, Germany). The 4D-MRA consisted of 13 sagittal acquisitions using a FLASH-3D-T1 sequence (TR 3.89 ms; TE 1.38 ms; flip angle, 17°; section thickness, 1 mm; FOV, 384 × 264 × 211 mm). In addition, sagittal T1 TSE, T2 TSE and T2 GRE sequences were obtained. The 4D-MRA examinations were reviewed by experienced neuroradiologists blinded to outcomes of the DSA and treatment.

### 2.3 Spinal angiography

Spinal angiography was performed by experienced neuro-interventionalists on a monoplane angiography system (Philips Medical Systems, Best, The Netherlands). Spinal arteries were selected using a 4-French headhunter catheter or Cobra C1/2 catheter (Terumo®) according to the decision of the interventionalist and were visualized after automatic injection of 6–8 ml of iodixanol (320 mg iodine/ml; Visipaque™, GE Healthcare, Cork, Ireland). Angiography was performed with a 178 or 229 mm field of view and a 1024 matrix, yielding a measured pixel size of 0.17 × 0.17 mm^2^ or 0.22 × 0.22 mm^2^, respectively.

### 2.4 Data analysis

Quantitative variables were described as means and standard deviations. Diagnostic performance measures of 4D-MRA were calculated (sensitivity, positive predictive value, specificity, and negative predictive value) with DSA serving as the reference standard.

## 3 Results

### 3.1 Retrospective cohort

Overall, 120 patients with who underwent 4D-MRA for the clinical suspicion of having a SDAVF were identified. Main indications for 4D-MRA were clinical signs of a transverse spinal cord lesion (n = 49), ectatic veins on conventional MRI (n = 22), posterior fossa or spinal subarachnoid hemorrhage (n = 21), or a combination of the preceding. No specific indication for spinal 4D-MRA was mentioned in the remaining examinations. The 4D-MRA examinations performed in patients who had a subarachnoid hemorrhage yielded a negative result in all cases. For the clinical suspicion of a transverse lesion of the spinal cord, 4D-MRA identified a SDAVF in 15 of the 49 cases. 4D-MRA performed for the presence of ectatic veins on previous spinal MRI yielded a SDAVF in 13 of the 22 cases.

In 90 patients no DSA and no follow-up was available after 4D-MRA, therefore we identified a subgroup of 30 patients (21 males, 9 females; mean age 56 years with a SD of 21 years) who have had both 4D-MRA and DSA (n = 22), or clinical/radiological follow-up (n = 8). It was confirmed that DSA was performed after 4D-MRA. The demographic data of the group are provided in Table 1.

**Table 1:** Demographics of the study group (n = 30) SDAVF: spinal dural arteriovenous fistula, DSA: digital subtraction angiography, 4D-MRA: time-resolved contrast enhanced MR angiography.

Patient characteristics (n = 30)	
Sex	
Male	21 (70%)
Female	9 (30%)
Age, years (SD)	56 (±21)
Time delay between 4D-MRA and DSA, days (SD)	40 (±83)
SDAVF identified on DSA	
Yes	19 (63%)
No	11 (37%)
Side of SDAVF on DSA	
Left	7 (37%)
Right	11 (58%)
Bilateral	1 (5%)
Treatment of SDAVF	
Endovascular	2 (9%)
Surgical	11 (50%)
Missing data	6 (27%)
Follow-up	
4D-MRA	15 (68%)
DSA	7 (32%)

### 3.2 Diagnostic accuracy of 4D-MRA

Results of the 4D-MRA and DSA examinations are provided in Table 2. In the group of 30 cases, 4D-MRA was positive for a SDAVF in 20 cases and 10 were read negative. On DSA, a SDAVF was found in 19 patients and 3 patients had a negative DSA. Therefore, one case was considered as a false positive read on 4D-MRA. The group of 8 patients with clinical/radiological follow-up showed to have no signs that a SDAVF was present, these patients were therefore considered to be true-negatives and were included in the statistical analysis.

**Table 2:** Results of spinal 4D-MRA and DSA examinations.

	DSA/clinical follow-up Positive	DSA/clinical follow-up Negative	Total
Outcome MRA Positive	19	1	20
Outcome MRA Negative	0	10	10
Total	19	11	30

Sensitivity of 4D-MRA to identify a SDAVF was 100%, positive predictive value of 95%, specificity was 91%, negative predictive value of 100%. In 19 subjects both 4D-MRA and DSA identified the presence of a SDAVF. These included eleven right-sided and seven left-sided SDAVFs as confirmed by DSA, and there was one case with bilateral AV-shunting. In 2 subjects, the level and sidedness of the SDAVF could not be identified on 4D-MRA, as was stated in the radiological report. The sidedness of the SDAVF was correctly identified on 4D-MRA in 14 of 19 subjects (74%), and the level of the SDAVF was correctly identified on 4D-MRA in 13 of 19 cases (68%).

## 4 Discussion

**Fig. 1:**
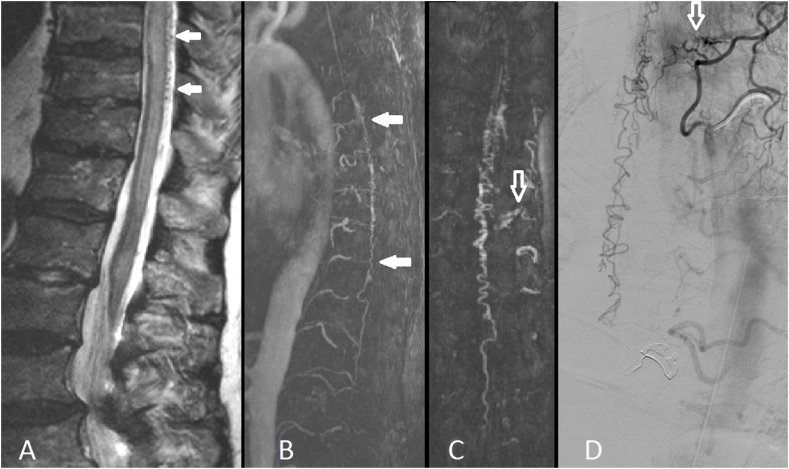
75-year-old male presenting with a SDAVF. Spinal MRI (A) demonstrates spinal cord edema with congested and dilated tortuous intradural veins surrounding the spinal cord. Additional 4D-MRA (B,C) showed a left sided SDAVF at the level of Th5-Th6. The SDAVF was confirmed by DSA (D).

The 4D-MRA technique relies on dynamic visualization of the spinal vasculature during the passing of a contrast agent, where arterio-venous shunting is detected by abnormal early enhancement of spinal veins (Backes and Nijenhuis, 2008), as demonstrated in Fig. 1. In this retrospective cohort study, the sensitivity of 4D-MRA for the diagnosis of a SDAVF was 100% and the specificity was 91% as compared to conventional angiography. This confirms that 4D-MRA has a high diagnostic accuracy for the detection of SDAVFs, which is in line with literature (Wójtowicz et al., 2023; Raymaekers et al., 2024).

The high negative predictive value of 4D-MRA, as demonstrated in both our study and existing literature, indicates that DSA may be unnecessary if spinal MRI and 4D-MRA show no evidence of a SDAVF. Conventional angiography should however still be considered in case of a positive, or inconclusive 4D-MRA, to confirm the presence and location of a SDAVF and to study its angio-architecture for treatment decision making. When a SDAVF is localized by 4D-MRA imaging, the angiography procedure can be tailored to the level of the suspected SDAVF, which reduces procedural time and lowers the risk of complications (Wójtowicz et al., 2023; Raymaekers et al., 2024). A previous study demonstrated that pre-angiographic 4D-MRA evaluation of SDAVF substantially reduces the radiation dose and volume of contrast agent associated with conventional spinal angiography (Luetmer et al., 2005).

Several limitations need to be addressed. Our series has a small sample size and it is important to note that there was a significant selection bias for DSA, as DSA was generally not performed in our institution when the 4D-MRA yielded a negative result. To enhance the sample size for statistical analysis, we aimed to include additional subjects, contingent upon the availability of clinical or radiological follow-up that confirmed these cases as true negatives. Due to the small number of true negatives in our cohort, slight uncertainty remains on the specificity and negative predictive value. However, it needs to be emphasized that clinical considerations and the findings on conventional spinal MRI should also be taken into account. Furthermore, the accuracy of the 4D-MRA study relies not only on the quality of the MRA acquisition but also on the expertise of the reader. In our study, both the 4D-MRA and DSA studies were assessed by experienced readers at a specialized tertiary referral center. Further limitations are the retrospective nature of the study, which leads to some missing data and may be subjected to verification bias.

## 5 Conclusion

The results indicate that 4D-MRA has a high sensitivity and specificity for the detection and localization of a SDAVF, which is in line with previous studies published in literature. Furthermore, 4D-MRA can serve to guide DSA and to shorten the procedural time, which reduces the risk of angiography related complications, and decreases patient discomfort.

## Declarations

This research did not receive any specific grant from funding agencies in the public, commercial, or not-for-profit sectors.

The authors have no relevant financial or non-financial interests to disclose.

All authors contributed to the study conception and design. Material preparation, data collection and analysis were performed by Sjoert A.H. Pegge, Frederick J.A. Meijer and Jeroen HD. Boogaarts. Literature search and manuscript preparation was performed by Vincent Raymaekers. All authors read and approved the final manuscript.

## Declaration of generative AI and AI-assisted technologies in the writing process

During the preparation of this work the author(s) used Microsoft Copilot in order to improve readability of some sentences. After using this tool/service, the author(s) reviewed and edited the content as needed and take(s) full responsibility for the content of the publication.

## Declaration of competing interest

The authors declare that they have no known competing financial interests or personal relationships that could have appeared to influence the work reported in this paper.
